# Investigating the prognostic significance of examined lymph node count in elderly women with cervical carcinoma: a SEER population-based study

**DOI:** 10.3389/fmed.2025.1619214

**Published:** 2025-11-25

**Authors:** Weilan Liu, Kaiyun Qin, Luyang Su, Xiaoyan Duan, Yan Jiang, Caifu Zhao, Wenbo Qi, Congwei Dai

**Affiliations:** 1 Center of Physical Examination, Hebei General Hospital, Shijiazhuang, Hebei, China; 2 Department of Gynecology, Hebei General Hospital, Shijiazhuang, Hebei, China; 3 Department of Obstetrics and Gynecology, The Second Hospital of Hebei Medical University, Shijiazhuang, Hebei, China

**Keywords:** cervical cancer, examined lymph nodes (ELNs), elderly patients, surgery, nomogram, overall survival (OS), prognosis

## Abstract

**Objective:**

This study aims to explore the prognostic factors including the number of examined lymph nodes (ELNs) for elderly cervical cancer patients who have undergone surgery, and to develop and validate a novel model to predict survival prognosis in this patient population.

**Methods:**

A database comprising patients aged 65 years or older diagnosed with stage I-IVA cervical cancer who subsequently underwent surgery was retrieved from the Surveillance, Epidemiology, and End Results Program (SEER). Cox regression analyses were conducted to examine the relationship between the number of ELNs and overall survival (OS). Propensity score matching(PSM) was conducted to control the influence of confounding factors and competitive risk analyses were used to evaluate the relationship between ELN and cervical cancer-specific mortality. A nomogram was constructed based on the training set and validated using the testing set.

**Results:**

Eight hundred and seven participants were included totally. Kaplan–Meier survival analysis demonstrated that patients with a higher number of ELNs had significantly prolonged OS. Cox regression analysis confirmed that the number of ELNs was an independent prognostic factor for OS. After PSM, the competitive risk analysis revealed no significant association between the number of ELNs and the risk of cervical cancer specific mortality. A predictive model incorporating variables including the ELNs count, age, FIGO staging, and radiotherapy status was developed, evaluated, and validated to predict survival rate. The model demonstrated high predictive accuracy for survival outcomes.

**Conclusion:**

The ELN count is a prognostic factor worth considering in elderly patients with cervical cancer and our survival rate prediction model integrating ELN count has good predictive ability and universality.

## 1 Introduction

Cervical carcinoma has become the second most common cancer among females globally. Although the incidence and mortality rates have gradually decreased in recent years due to widespread screening programs, this disease remains a significant threat to women’s health, particularly in less developed countries (1). In China, there were 111,820 new cases of cervical cancer reported in 2022, with an estimated 61,579 deaths attributed to the disease (2). This substantial burden on public health highlights the need for continued efforts in prevention and treatment. Younger women are the primary beneficiaries of cervical cancer screening, while elderly women over 65 years old may neglect regular screenings and physical examinations (3). Consequently, there is a second peak in incidence between the ages of 60 and 70, likely due to declining immunity. Additionally, the incidence of cancer in the residual cervix following subtotal hysterectomy may be underestimated in older women (4). Studies have shown that women over 65 diagnosed with cervical cancer are more likely to receive chemoradiotherapy rather than surgery, resulting in shorter overall survival compared to younger patients (3, 5). Herefore, it is crucial to explore the treatment and prognosis of elderly cervical cancer patients.

Lymph node status plays a critical role in tumor progression and prognosis by contributing to accurate FIGO staging (6). Pelvic lymphadenectomy combined with radical hysterectomy is an essential procedure for treating early-stage cervical cancer (7). Several studies have shown that a higher number of ELNs correlates with improved survival rates in esophageal (8), colorectal (9),gastric (10) and endometrial cancers (11). Similar findings have been reported in cervical cancer (12). However, lymph node dissection can also lead to increased postoperative complications, such as lower extremity lymphedema and pelvic lymphocysts (13). The predictive value of ELNs on the prognosis of elderly patients with cervical cancer who undergo surgery remains unclear, and the optimal number of lymph nodes to be excised is still debated.

To address these issues, we utilized data from the SEER program of the United States National Cancer Institute to conduct a population-based retrospective analysis investigating the relationship between ELNs and overall survival in elderly patients with cervical cancer. Additionally, we developed a predictive model incorporating ELNs and other relevant variables to estimate patient prognosis.

## 2 Methods

**Figure 1:**
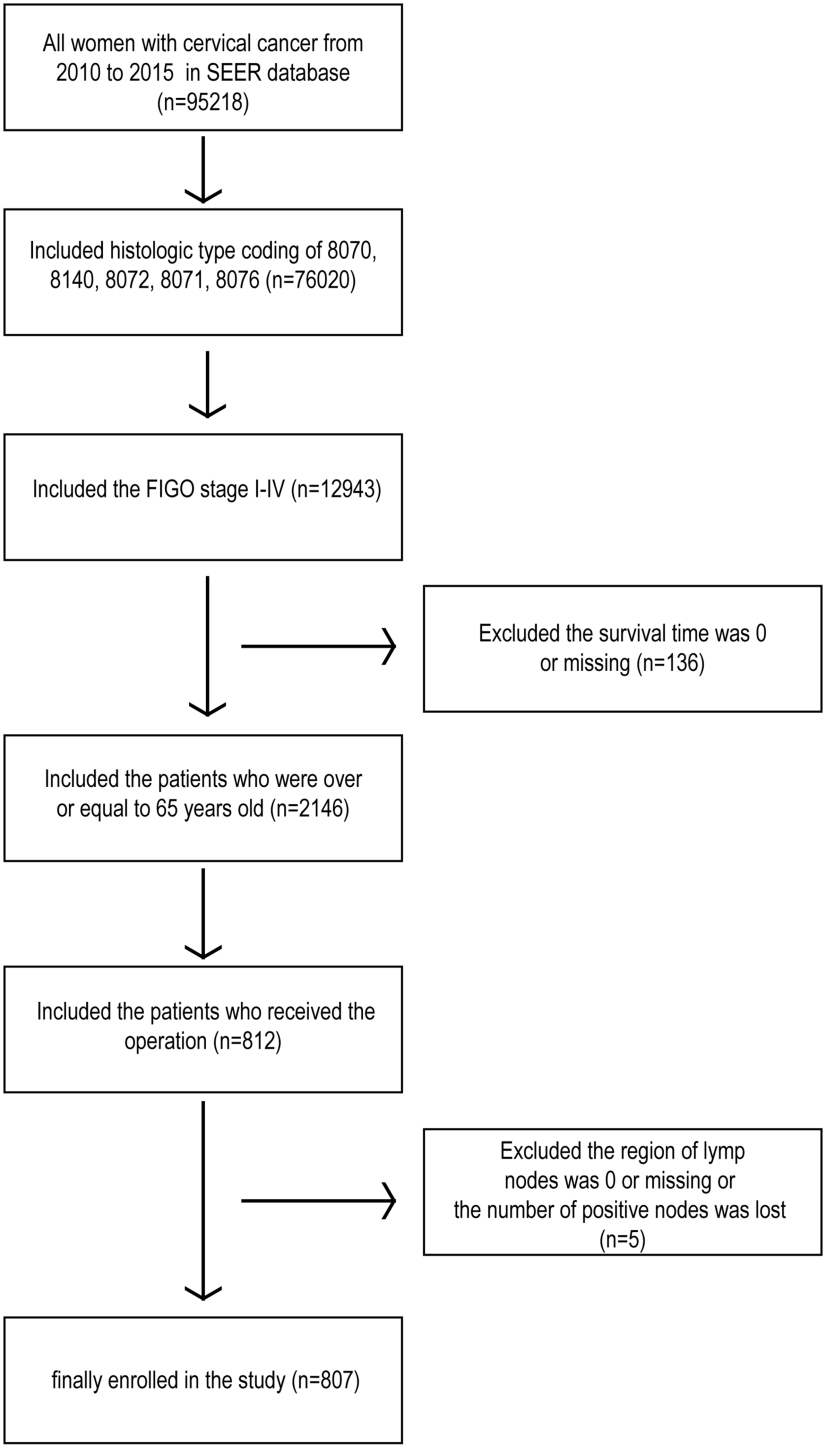
Flowchart of the inclusion and exclusion criteria of the participants.

The data of patients with cervical cancer were retrieved from the SEER Program database of the United States National Cancer Institute using SEER*Stat software (version 8.3.8). The dataset encompassed all patients diagnosed with stage I-IV cervical cancer who underwent surgical treatment between 2010 and 2015. Additional inclusion criteria included: patients aged 65 years or older; histologic type codes 8,070, 8,140, 8,072, 8,071, and 8,076; and primary site limited to the cervix uteri. Patients with incomplete information on survival time, lymph node region, or positive lymph node count were excluded (Figure 1). As the data were publicly available and did not involve patient privacy, ethics review board approval or informed consent from patients was not required.

Patient characteristics including age, race, marital status, diagnosis date, FIGO grade, surgical approaches, number of ELNs, chemotherapy or radiotherapy administration, histological type, pathological grading, and presence of multiple lesions were extracted from the SEER database. The cutoff points for the number of ELNs were determined using the surviMisc package in R software, categorizing ELNs into three groups (0, 1–15, 16–56). Continuous variables such as age were converted into categorical variables based on their frequency distribution, while other variables like marital status and FIGO stage were also categorized accordingly. Overall survival (OS), defined as the time interval from diagnosis to death from any cause, was used as the primary endpoint to evaluate prognosis.

### 2.1 Statistical analysis

Clinical characteristics were summarized for the overall cohort stratified by three distinct ELN groups. Frequencies and percentages were calculated for all demographic and clinical variables. The Kaplan–Meier curves illustrated OS across the different groups, with the log-rank test used to assess statistical significance. Univariate Cox proportional hazards regression was conducted to analyze correlations and identify potential prognostic factors. Subsequently, multivariate Cox regression analysis, adjusted for confounding variables, was performed to determine independent predictors of patient OS.

A multinomial logistic regression model was constructed with ELN group as the dependent variable and the covariates as independent variables to calculate propensity scores. Subsequently, 1:1:1 propensity score matching was performed across groups to minimize differences in covariate distributions. After matching, covariate balance was assessed using standardized mean differences (SMD), with an SMD < 0.1 considered indicative of well-balanced covariates. The matched data is used for subsequent single-factor and multi-factor analyses based on the competitive risk model. In the competing risk analysis, survival cases were coded as 0, deaths from cervical cancer as 1, and deaths from non-cervical cancer as 2, with the latter being considered a competing event. The Fine and Gray competing risks model was utilized to estimate the subdistribution hazard ratios (SHRs) and their corresponding 95% confidence intervals (CIs), thereby analyzing the association between each variable and the outcome events. The cumulative incidence of the outcome variables was calculated using the cumulative incidence function (CIF), and CIF curves for covariates were plotted. Gray’s test was employed to evaluate differences in cumulative incidence among distinct ELNs groups.

All patients in the datasets were randomly allocated into training and validation cohorts at a 1:1 ratio. Univariate and multivariate regression analyses based on the Cox proportional hazards model were conducted using the training cohort. The variables were screened using the Stepwise method, and a Nomogram was constructed to predict the 12-, 36-, and 60-month survival rates in patients with cervical cancer. The C-index and the area under the receiver operating characteristic (ROC) curve (AUC) were utilized to evaluate the model’s predictive performance. Additionally, the calibration curve was employed to assess the agreement between predicted and actual outcomes of the Cox model, thereby visualizing the accuracy of the model. Brier scores were utilized to comprehensively assess the discrimination and calibration of the model. Decision curve analysis (DCA) was employed to evaluate the clinical utility of the novel prediction model. All statistical analyses were conducted using R software (version 4.0.5, released on 2021-03-31)^1^. A two-sided *p* < 0.05 was considered statistically significant.

## 3 Results

In total, 95,218 cervical cancer patients were identified in the SEER database from 2010 to 2015, of whom 807 cases met the inclusion criteria. The clinicopathological characteristics of these patients are summarized in Table 1. Among the 807 patients, 369 (45.7%) had no lymph nodes examined, 228 (28.3%) had 1 to 15 examined lymph nodes (ELNs), and 210 (26.0%) had 16 to 56 ELNs. As shown in Table 1, significant differences were observed in age, race, histological type, and cancer grade among the three ELNs groups (*p* < 0.05). In contrast, no significant differences were found in marital status, time of diagnosis, chemotherapy or radiotherapy administration, presence of multiple lesions, and FIGO staging among the groups (*p* > 0.05).

**Table 1:** Baseline of clinicopathological characteristics and association analysis of the number of examined lymph nodes and other clinical factors.

Variable	Total (*n* = 807, %)	ELN 0 (*n* = 369, %)	ELN 1–15 (*n* = 228, %)	ELN 16–56 (*n* = 210, %)	*p*
Age					<0.001
65–70	415 (51.4)	170 (46.1)	116 (50.9)	129 (61.4)	
71–80	284 (35.2)	133 (36)	84 (36.8)	67 (31.9)	
81	108 (13.4)	66 (17.9)	28 (12.3)	14 (6.7)	
Race					<0.001
Black	98 (12.1)	65 (17.6)	25 (11)	8 (3.8)	
Others	107 (13.3)	40 (10.8)	36 (15.8)	31 (14.8)	
White	602 (74.6)	264 (71.5)	167 (73.2)	171 (81.4)	
Marital status					0.095
Divorced/Separated	140 (17.3)	58 (15.7)	40 (17.5)	42 (20)	
Married	277 (34.3)	112 (30.4)	84 (36.8)	81 (38.6)	
Single/Unmarried	114 (14.1)	53 (14.4)	33 (14.5)	28 (13.3)	
Widowed/Others	276 (34.2)	146 (39.6)	71 (31.1)	59 (28.1)	
Years of diagnosis					0.388
2010–2012	436 (54)	194 (52.6)	120 (52.6)	122 (58.1)	
2013–2015	371 (46)	175 (47.4)	108 (47.4)	88 (41.9)	
Radiotherapy					0.133
No/Unknown	429 (53.2)	182 (49.3)	128 (56.1)	119 (56.7)	
Yes	378 (46.8)	187 (50.7)	100 (43.9)	91 (43.3)	
Chemotherapy					0.226
No	541 (67)	239 (64.8)	163 (71.5)	139 (66.2)	
Yes	266 (33)	130 (35.2)	65 (28.5)	71 (33.8)	
FIGO stage					0.171
I	543 (67.3)	260 (70.5)	148 (64.9)	135 (64.3)	
II	141 (17.5)	64 (17.3)	43 (18.9)	34 (16.2)	
III–IV	123 (15.2)	45 (12.2)	37 (16.2)	41 (19.5)	
Number of lesions					0.195
1st	599 (74.2)	263 (71.3)	173 (75.9)	163 (77.6)	
*N* ≥ 2	208 (25.8)	106 (28.7)	55 (24.1)	47 (22.4)	
Grade					<0.001
I–II	425 (52.7)	190 (51.5)	119 (52.2)	116 (55.2)	
III–IV	260 (32.2)	99 (26.8)	87 (38.2)	74 (35.2)	
Unknown	122 (15.1)	80 (21.7)	22 (9.6)	20 (9.5)	
Histological type					<0.001
AC	133 (16.5)	41 (11.1)	44 (19.3)	48 (22.9)	
SCC	674 (83.5)	328 (88.9)	184 (80.7)	162 (77.1)	
Number of ELNs					<0.001
0	369 (45.7)	369 (100)	0 (0)	0 (0)	
1–15	228 (28.3)	0 (0)	228 (100)	0 (0)	
16–56	210 (26)	0 (0)	0 (0)	210 (100)	

AC, adenomatous carcinoma; SCC, squamous carcinoma; ELNs, examined lymph nodes.

**Figure 2:**
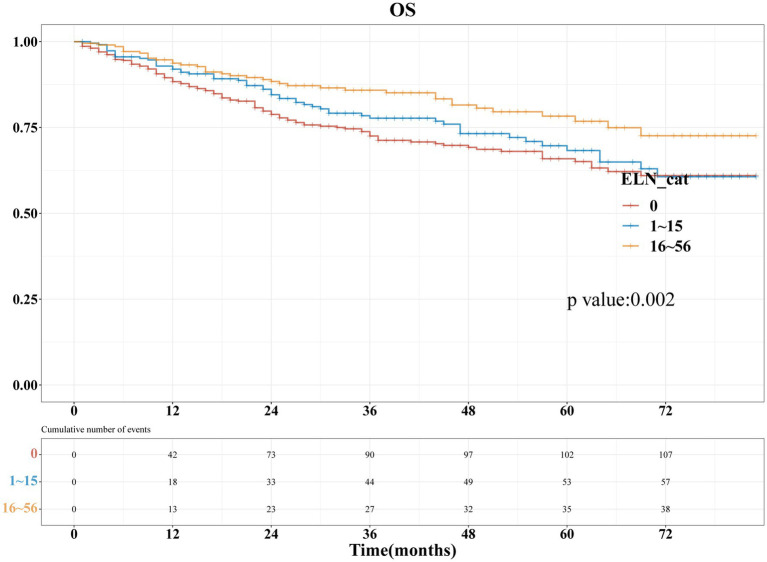
Kaplan–Meier survival curves were utilized to illustrate the changes in overall survival (OS) rates across different ELN groups.

**Figure 3:**
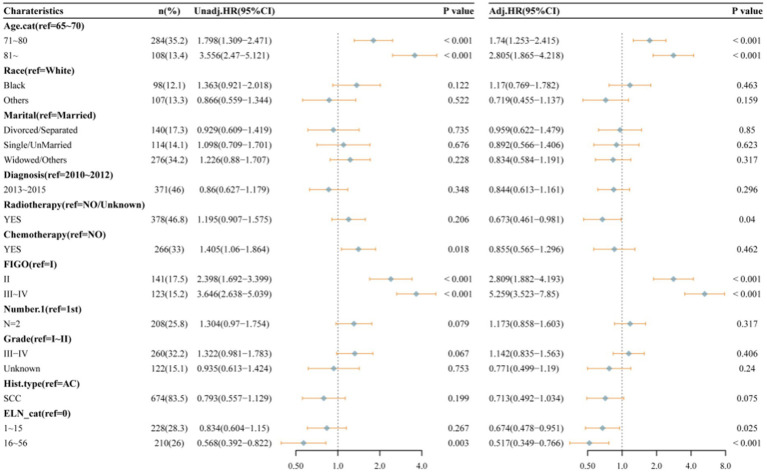
Univariate and multivariable Cox proportional hazard regression analyses of factors associated with overall survival of all patients.

Figure 2 presents the Kaplan–Meier analyses of the three ELNs groups, demonstrating that patients with a higher number of ELNs had significantly longer overall survival (OS, *p* = 0.002). To further investigate the impact of ELNs on patient survival, univariate and multivariate Cox regression analyses, as well as subsequent multivariate competing risk analyses, were conducted. As illustrated in Figure 3, univariate factor analysis revealed a significant correlation between 16 and 56 ELNs and changes in OS (HR = 0.568; 95% CI: 0.392–0.822; *p* = 0.003), whereas 1–15 ELNs did not show statistical significance (HR = 0.834; 95% CI: 0.604–1.15; *p* = 0.267). After adjusting for multiple factors, multivariate analysis confirmed that 1–15 ELNs were significantly associated with improved OS (HR = 0.674; 95% CI: 0.478–0.951; *p* = 0.025), and 16–56 ELNs remained independently associated with better OS (HR = 0.517; 95% CI: 0.349–0.766; *p* < 0.001). Additionally, age, receipt of radiotherapy, and FIGO stage were also identified as independent predictors of overall survival in the multivariate analysis.

**Figure 4:**
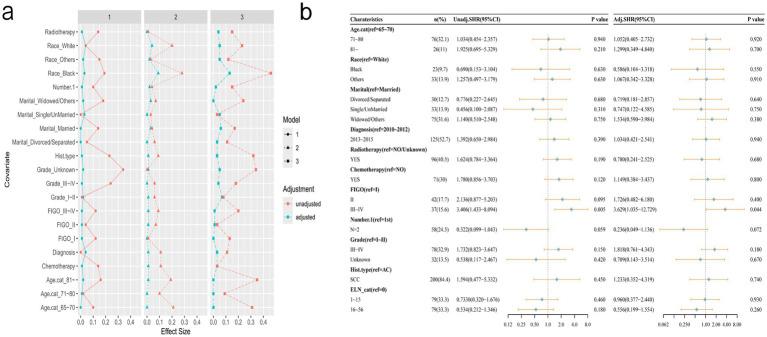
After multicategory propensity score matching, covariate balance was assessed using standardized mean differences (SMD) **(a)**, followed by univariate and multivariate analyses based on a competing risk model **(b)**.

**Figure 5:**
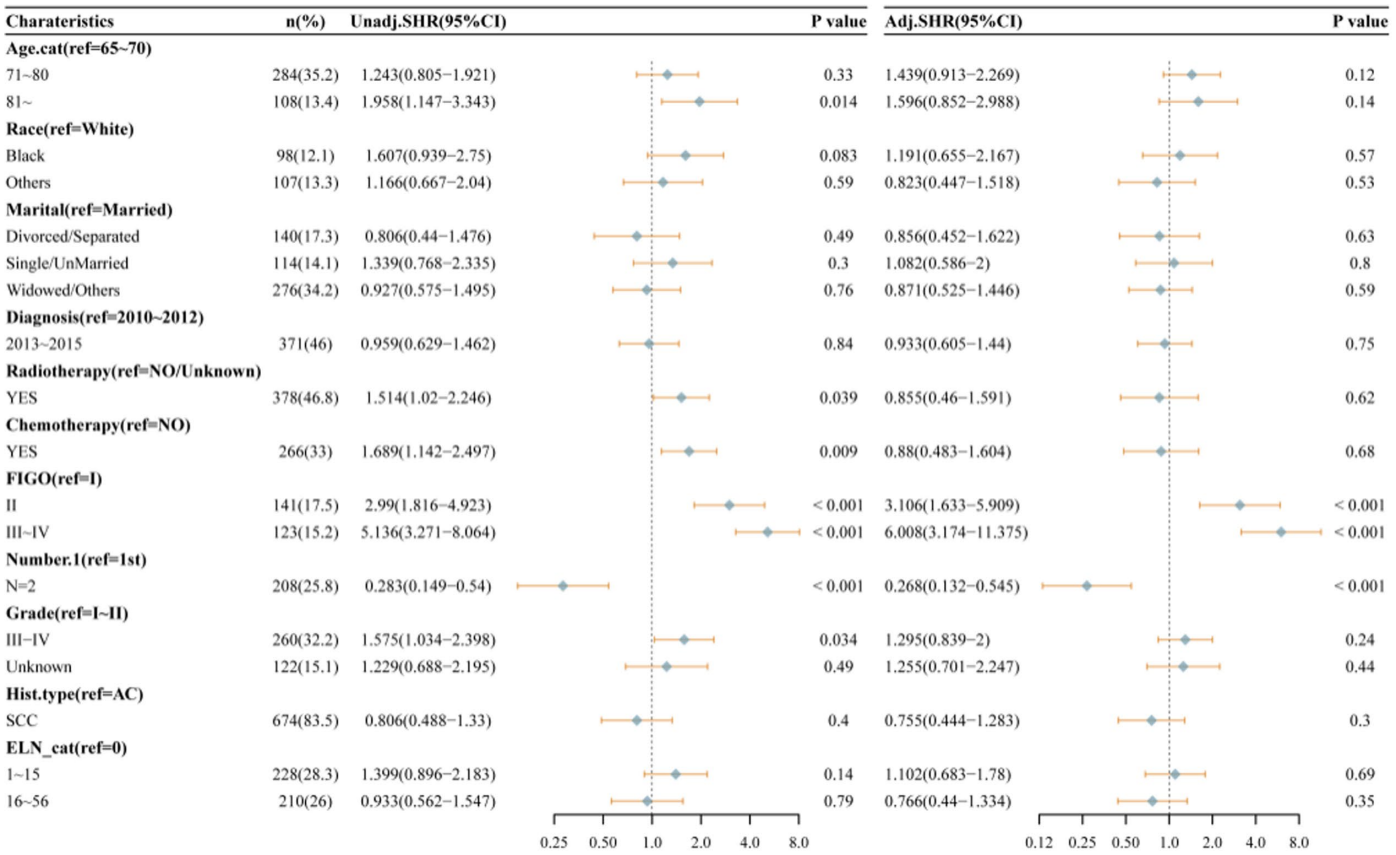
Multivariate competitive risk analysis of factors associated with overall survival of all patients.

As illustrated in Figure 4, following propensity score matching (PSM) processing, the final analytic sample comprised 237 cases. No statistically significant differences in any of the covariates were observed between the groups (Table 2). The following multivariate competitive risk analysis using the Fine & Gray competitive risk model revealed no significant association between changes in the risk of death from cervical cancer and the number of ELNs (ELN 1–15 vs. ELN 0: *p* = 0.93; ELN 16–56 vs. ELN 0: *p* = 0.26). Next, a full-sample competitive risk analysis was conducted. Specifically, for patients with 1–15 ELNs compared to those with 0 ELNs, the subhazard ratio (SHR) was 1.102 (95% CI: 0.683–1.78; *p* = 0.69). Similarly, for patients with 16–56 ELNs compared to those with 0 ELNs, the SHR was 0.766 (95% CI: 0.44–1.334; *p* = 0.35) (Figure 5).

**Table 2:** The characteristics analyses after propensity score matching.

Variables	Total (*n* = 237, %)	ELN 0 (*n* = 79, %)	ELN 1–15 (*n* = 79, %)	ELN 16–56 (*n* = 79, %)	*p*
Age					0.877
65 ~ 70	135 (57)	43 (54.4)	47 (59.5)	45 (57)	
71 ~ 80	76 (32.1)	25 (31.6)	25 (31.6)	26 (32.9)	
81	26 (11)	11 (13.9)	7 (8.9)	8 (10.1)	
Race					0.989
Black	23 (9.7)	7 (8.9)	8 (10.1)	8 (10.1)	
Others	33 (13.9)	11 (13.9)	12 (15.2)	10 (12.7)	
White	181 (76.4)	61 (77.2)	59 (74.7)	61 (77.2)	
Marital status					0.666
Divorced/Separated	30 (12.7)	9 (11.4)	11 (13.9)	10 (12.7)	
Married	99 (41.8)	31 (39.2)	34 (43)	34 (43)	
Single/Unmarried	33 (13.9)	16 (20.3)	8 (10.1)	9 (11.4)	
Widowed/Others	75 (31.6)	23 (29.1)	26 (32.9)	26 (32.9)	
Years of diagnosis					0.214
2010 ~ 2012	112 (47.3)	31 (39.2)	41 (51.9)	40 (50.6)	
2013 ~ 2015	125 (52.7)	48 (60.8)	38 (48.1)	39 (49.4)	
Radiotherapy					0.854
No/Unknown	141 (59.5)	48 (60.8)	45 (57)	48 (60.8)	
Yes	96 (40.5)	31 (39.2)	34 (43)	31 (39.2)	
Chemotherapy					0.770
No	166 (70)	53 (67.1)	57 (72.2)	56 (70.9)	
Yes	71 (30)	26 (32.9)	22 (27.8)	23 (29.1)	
FIGO stage					0.878
I	158 (66.7)	49 (62)	55 (69.6)	54 (68.4)	
II	42 (17.7)	16 (20.3)	13 (16.5)	13 (16.5)	
III ~ IV	37 (15.6)	14 (17.7)	11 (13.9)	12 (15.2)	
Number of lesions					0.493
1st	179 (75.5)	62 (78.5)	56 (70.9)	61 (77.2)	
*N* ≥ 2	58 (24.5)	17 (21.5)	23 (29.1)	18 (22.8)	
Grade					0.633
I ~ II	127 (53.6)	43 (54.4)	46 (58.2)	38 (48.1)	
III–IV	78 (32.9)	27 (34.2)	24 (30.4)	27 (34.2)	
Unknown	32 (13.5)	9 (11.4)	9 (11.4)	14 (17.7)	
Histological type					0.659
AC	37 (15.6)	10 (12.7)	13 (16.5)	14 (17.7)	
SCC	200 (84.4)	69 (87.3)	66 (83.5)	65 (82.3)	

AC, adenomatous carcinoma; SCC, squamous carcinoma; ELNs, examined lymph nodes.

**Figure 6:**
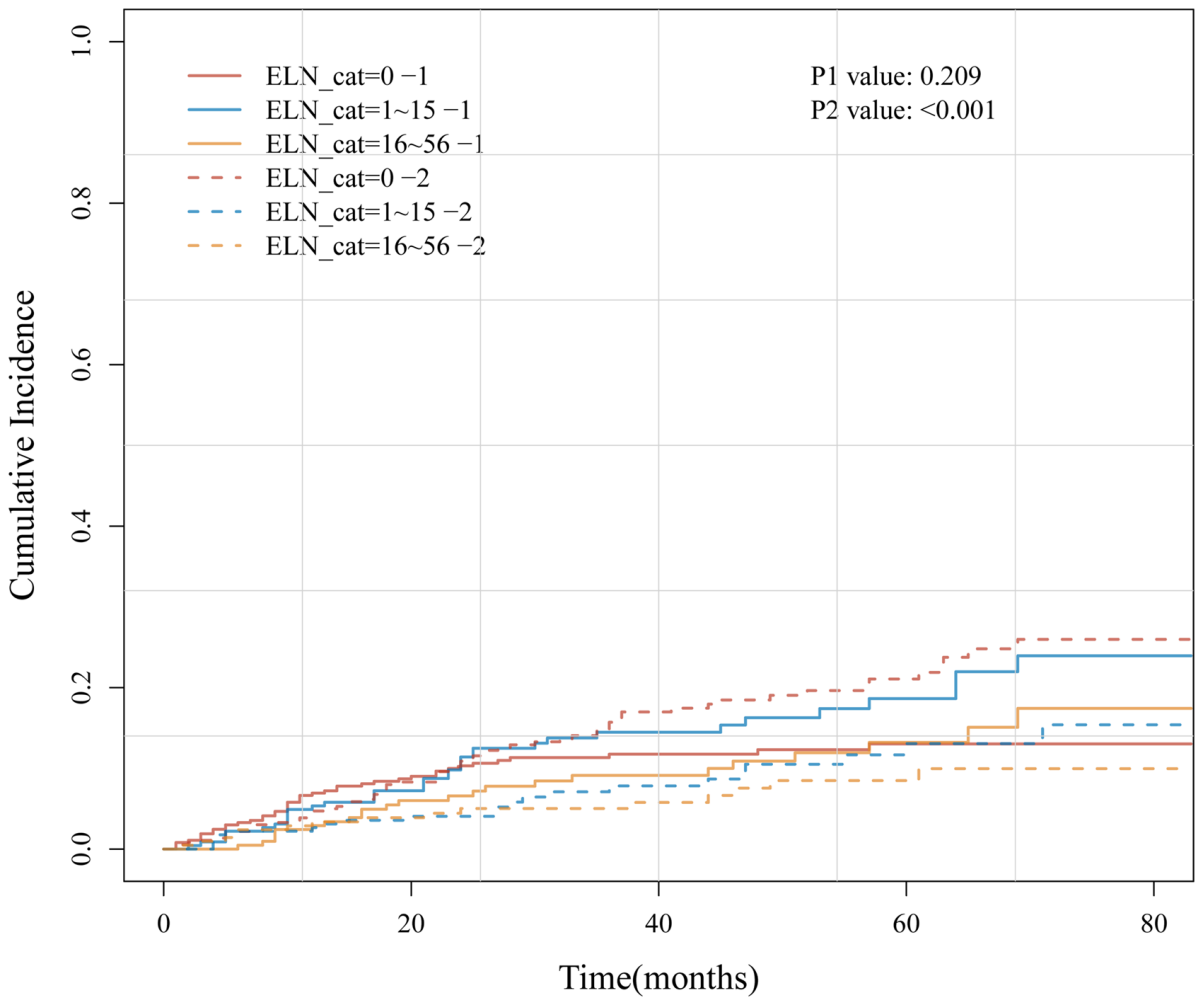
The CIF curve of the cumulative risk of cervical cancer-specific mortality (1) and the cumulative risk of non-cervical cancer death (2) in three groups of ELN.

Figure 6 presents the cumulative incidence function (CIF) curves for the three groups of ELNs. Univariate Gray’s test indicated that an increase in the number of ELNs was not significantly associated with an increased cumulative risk of cervical cancer-specific mortality (*p* = 0.209), but it was significantly associated with the cumulative risk of non-cervical cancer death (competing event) (*p* < 0.001).

**Figure 7:**
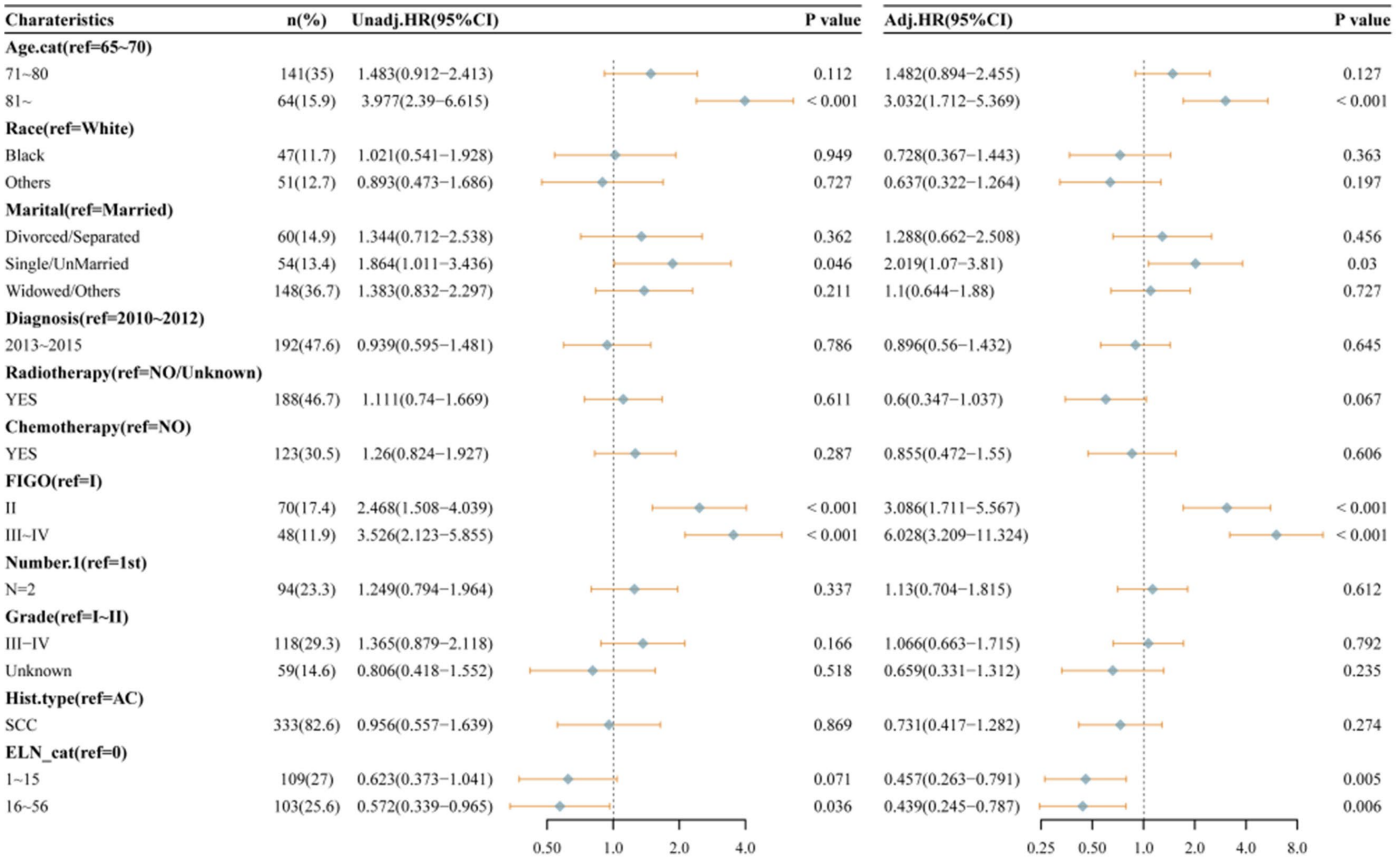
Univariate and multivariable Cox proportional hazard regression analyses of factors associated with overall survival of patients in training set.

**Figure 8:**
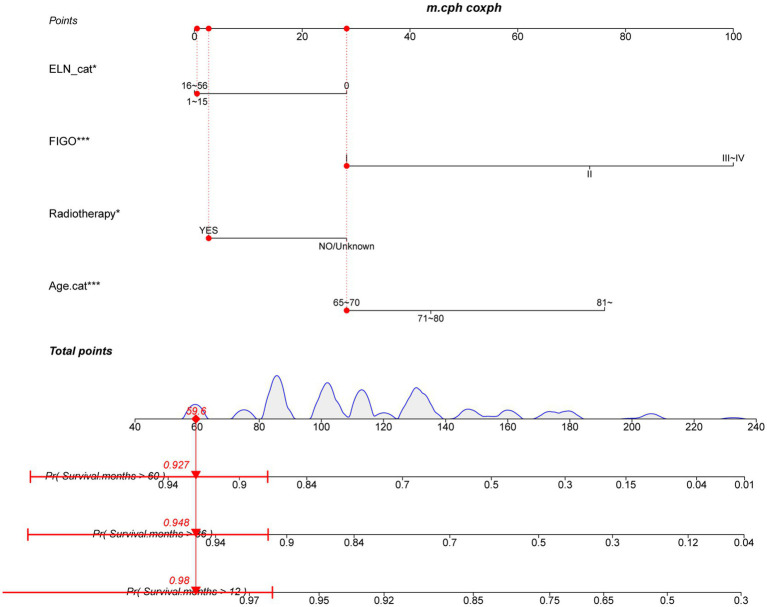
A nomogram to predict the survival rate of the training set.

**Figure 9:**
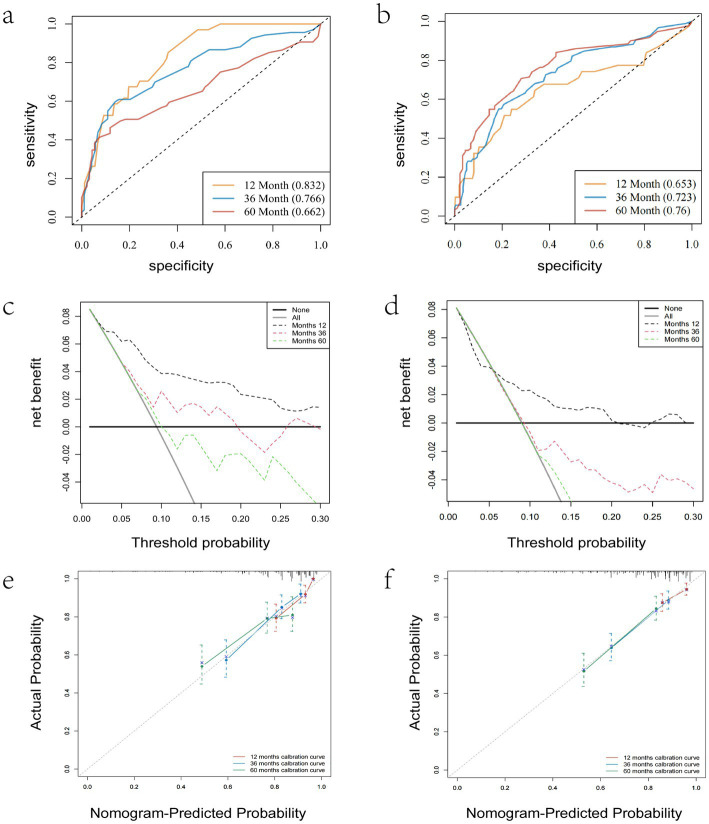
ROC curves **(a,b)**, Calibration curves **(c,d)** and decision curve analysis **(e,f)** were used to evaluate the prediction performance of the nomogram to predict the survival rate. **(a,c,e)** For training validation; **(b,d,f)** for external verification.

As illustrated in Table 3, the dataset was randomly and evenly partitioned into a training set and a validation set. Initially, Cox regression analysis was performed on the training set. Following multivariate adjustment, both the 1–15 ELNs group and the 16–56 ELNs group exhibited a significant association with improved OS. Specifically, for the 1–15 ELNs group, the hazard ratio (HR) was 0.457 (95% CI: 0.263–0.791, *p* = 0.005), while for the 16–56 ELNs group, the HR was 0.439 (95% CI: 0.245–0.787, *p* = 0.006) (Figure 7). Figure 8 illustrates a nomogram constructed using the regplot package in R for predicting survival rates. This nomogram integrates several prognostic factors, including the number of ENLs, patient age, FIGO stage, and the administration of radiotherapy, which were selected via stepwise regression analysis. To evaluate the model’s discriminatory performance, time-dependent ROC curves were generated based on the training set, yielding AUC values of 83.2% at 12 months, 76.6% at 36 months, and 62.2% at 60 months. The C-index and the Brier score at 12, 36 and 60 months indicated that the model exhibited substantial predictive accuracy and calibration. Furthermore, the decision curve analysis (DCA) confirmed significant clinical utility. Furthermore, the calibration curve exhibited a high degree of alignment with the standard curve, thereby validating the model’s accuracy (Figures 9a,c,e).

**Table 3:** The characteristics of the train set and the validation set.

Variable	Total (*n* = 807, %)	Training set (*n* = 403, %)	Validation set (*n* = 404, %)	*p*
Age				0.101
65–70	415 (51.4)	198 (49.1)	217 (53.7)	
71–80	284 (35.2)	141 (35)	143 (35.4)	
81	108 (13.4)	64 (15.9)	44 (10.9)	
Race				0.778
Black	602 (74.6)	305 (75.7)	297 (73.5)	
Others	98 (12.1)	47 (11.7)	51 (12.6)	
White	107 (13.3)	51 (12.7)	56 (13.9)	
Marital status				0.194
Divorced/Separated	277 (34.3)	141 (35)	136 (33.7)	
Married	140 (17.3)	60 (14.9)	80 (19.8)	
Single/Unmarried	114 (14.1)	54 (13.4)	60 (14.9)	
Widowed/Others	276 (34.2)	148 (36.7)	128 (31.7)	
Years of diagnosis				0.379
2010–2012	436 (54)	211 (52.4)	225 (55.7)	
2013–2015	371 (46)	192 (47.6)	179 (44.3)	
Radiotherapy				0.97
No/Unknown	429 (53.2)	215 (53.3)	214 (53)	
Yes	378 (46.8)	188 (46.7)	190 (47)	
Chemotherapy				0.162
No	541 (67)	280 (69.5)	261 (64.6)	
Yes	266 (33)	123 (30.5)	143 (35.4)	
FIGO stage				0.026
I	543 (67.3)	285 (70.7)	258 (63.9)	
II	141 (17.5)	70 (17.4)	71 (17.6)	
III–IV	123 (15.2)	48 (11.9)	75 (18.6)	
Number of lesions				0.131
1st	599 (74.2)	309 (76.7)	290 (71.8)	
*N* ≥ 2	208 (25.8)	94 (23.3)	114 (28.2)	
Grade				0.131
I–II	425 (52.7)	226 (56.1)	199 (49.3)	
III–IV	260 (32.2)	118 (29.3)	142 (35.1)	
Unknown	122 (15.1)	59 (14.6)	63 (15.6)	
Histological type				0.559
AC	133 (16.5)	70 (17.4)	63 (15.6)	
SCC	674 (83.5)	333 (82.6)	341 (84.4)	
Number of ELNs				0.615
0	369 (45.7)	191 (47.4)	178 (44.1)	
1–15	228 (28.3)	109 (27)	119 (29.5)	
16–56	210 (26)	103 (25.6)	107 (26.5)	

AC, adenomatous carcinoma; SCC, squamous carcinoma; ELNs, examined lymph nodes.

**Figure 10:**
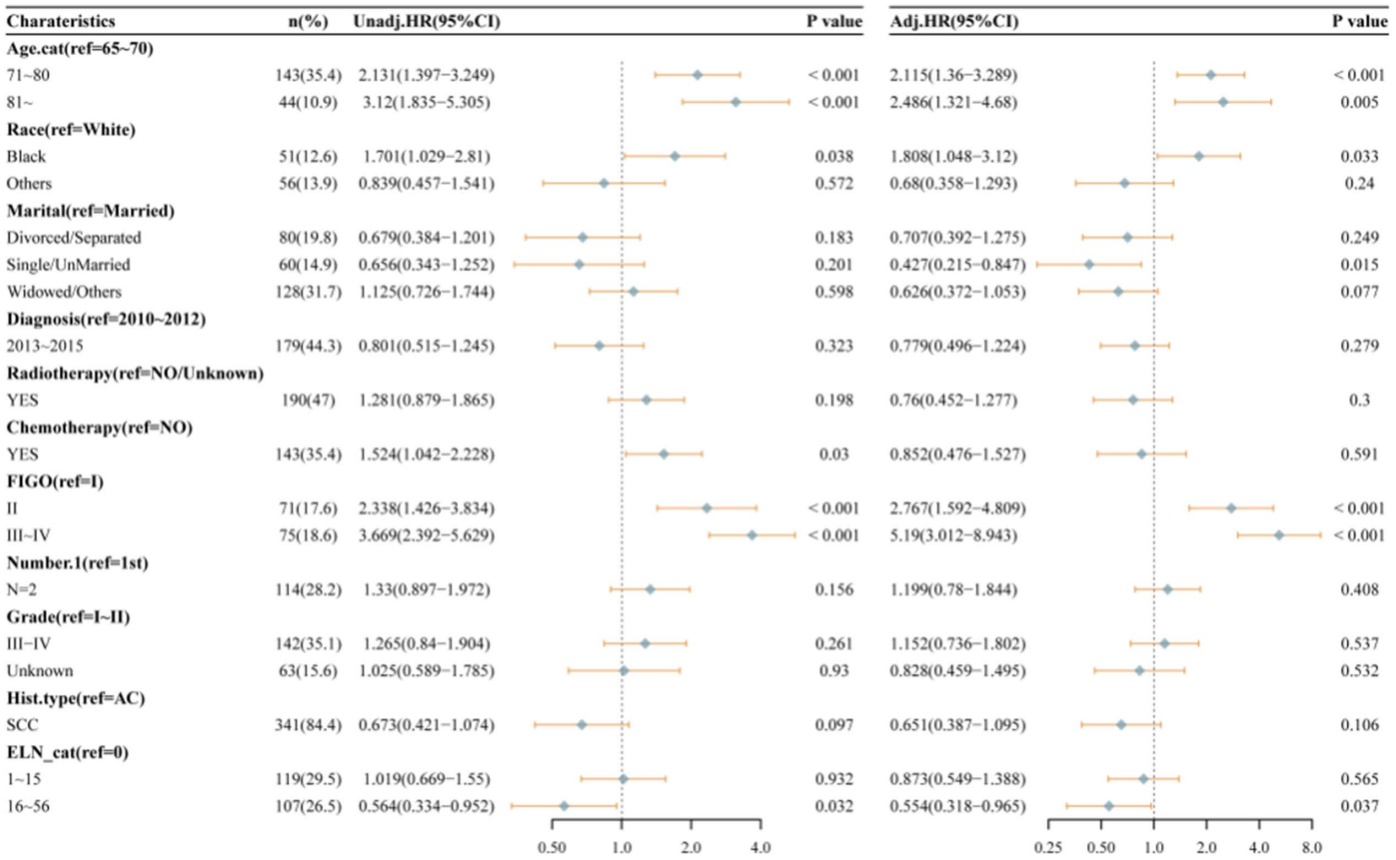
Univariate and multivariable Cox proportional hazard regression analyses of factors associated with overall survival of patients in validation set.

Figure 10 presents the forest plot of the validation set after conducting univariate and multivariable Cox proportional hazards regression analyses. After adjusting for multiple variables, patients with 16–56 ELNs demonstrated a significantly higher OS compared to those in the 0 ELN group (HR = 0.554; 95% CI = 0.318–0.965; *p* = 0.037). This finding was consistent with the results from the training set. As shown in Figures 9b,d,f, the AUC values were 65.3% at 12 monthes, 72.3% at 36 months, and 76.0% at 60 months, The C-index and Brier score at 12 months, 36 months, and 60 months confirmed the high accuracy of the prediction model, and the DCA analysis results indicated a good clinical benefit. The calibration curve closely aligned with the ideal curve, suggesting high model accuracy.

## 4 Discussion

Due to delayed screening of elderly cervical cancer patients, the cancer is often diagnosed at a more advanced stage. This delay is exacerbated by their reduced physical tolerance and higher susceptibility to complications. Economic limitations and poor physical conditions further contribute to a worse prognosis and increased mortality rates (14). However, there is a paucity of research specifically addressing cervical cancer in elderly populations. The screening and clinical management of cervical cancer in older women pose significant challenges due to their generally poorer baseline health conditions and lower tolerance for surgery. Consequently, non-surgical treatments such as radiotherapy and chemotherapy are often preferred. Potential complications from surgery and pelvic lymphadenectomy represent critical considerations. Elisabeth et al. reported that only 38% of cervical cancer patients aged 65 and older chose surgical treatment, with merely 41% undergoing pelvic lymphadenectomy. Their study concluded that patients who did not undergo surgery had lower overall survival rates (15). Several studies have shown that elderly patients exhibit comparable tolerance to surgery, chemotherapy, and radiation therapy relative to younger patients (16, 17). Moreover, prior research examining the relationship between ELNs and cervical cancer prognosis has primarily concentrated on variations across different FIGO stages and histological subtypes, rather than considering age as a critical factor (12, 18). Besides, there is a paucity of studies specifically examining the relationship between ELNs and the survival rates of elderly cervical cancer patients aged 65 years and older. Consequently, it is imperative to assess the potential benefits of pelvic lymph node dissection in elderly patients diagnosed with cervical cancer.

In this research, we investigated the prognostic importance of ELNs count in cervical cancer patients who are 65 years of age or older using a population-based analysis approach. Our results indicate that the number of ELNs, age, FIGO stage, and the administration of radiotherapy are prognostic factors for overall survival. A nomogram was constructed based on these prognostic factors to predict the 12-, 36-, and 60-month overall survival rates following surgery in elderly patients with cervical cancer. After rigorous verification and comprehensive evaluation, the nomogram demonstrates the accurate predictive performance for survival rate and significant clinical utility.

Overall survival rate (OS) is widely regarded as the “gold standard” endpoint for evaluating the efficacy of pharmaceuticals, surgical procedures, and other therapeutic interventions, providing an objective measure of patients’ actual survival benefits (6, 19). Given the critical role of lymphatic involvement in prognosis evaluation, the FIGO staging system has incorporated lymphatic status since 2018 (6). Two survival benefits are associated with lymph node dissection: the direct survival benefit from removing macroscopically metastatic lymph nodes and the indirect survival benefit derived from accurate disease staging and personalized adjuvant therapy. Therefore, precise evaluation of lymph nodes is essential for guiding carcinoma treatment (20). Lim et al. reported that patients with cervical cancer and tumors larger than 4 cm exhibited significantly improved survival rates when the number of ELNs exceeded 40 (21). Koray et al. indicated that the lymph node ratio (LNR) serves as an independent prognostic factor for both disease-free survival (DFS) and OS in patients with advanced cervical cancer (22). Additionally, several studies have consistently demonstrated that the progression-free survival (PFS) of cervical cancer patients, especially those with positive lymph nodes, is influenced by the number of ELNs examined (23–25). These results are consistent with our findings regarding elderly patients. However, other studies have reported that the number of ELNs does not significantly influence survival outcomes. For instance, Ditto et al. concluded that a more extensive lymphadenectomy did not affect survival rates in patients with positive lymph nodes (LNs). Conversely, for patients with negative LNs, a higher ELN count was associated with improved survival outcomes (26). In contrast, Mao et al.’s study, which included 359 patients with FIGO stage IA-IIB cervical cancer and negative LNs, revealed that the number of ELNs was not an independent prognostic factor for early-stage node-negative cervical cancer (27).

The impact of ELN on the prognosis of cervical cancer remains highly debated. The discrepancies in outcomes can primarily be attributed to three factors. First, there is significant variability in the extent of lymph node dissection among patients. Second, inconsistencies in cancer staging and pathological types across studies further contribute to these discrepancies. Third, differences in patient baseline conditions and surgeons’ experience and skills also play a crucial role. In addition to surgical techniques, several other factors influence the number of ELNs retrieved. These include the methodology used for lymph node submission, pathologists’ performance and interpretation, physiological variations among patients, and the inclusion of diverse pathological types. Consequently, analyses of the impact of varying numbers of removed lymph nodes may be susceptible to bias.

Multivariable competing risks analysis plays a crucial and indispensable role in cancer prognosis, especially when multiple potential causes of death (competing events) are considered. By controlling for other confounding factors, the true effect of a specific factor can be more precisely evaluated (28). To evaluate the influence of potential unmeasured confounding on our conclusions, we calculated the E-value. The results showed that the E-value for the association between ELN and OS was 2.53, whereas the hazard ratio (HR) for age in the multivariate model was only 1.74 (Supplementary Figure S1). According to the interpretation criteria proposed by scholars such as VanderWeele and Ding (29, 30), an unmeasured confounder would need to be more strongly associated with both the exposure and the outcome than the E-value to fully explain or negate the observed association. Thus, the E-value in our study is notably higher than the impact strength of age, indicating that age alone is insufficient to fully account for the association between ELN and OS. In our study, the PSM was used as a robustness check to verify whether the main results were affected by confounding factors. The competitive risk analysis after PSM showed that the number of ELNs was not significantly associated with the risk of cervical cancer-specific death, which was consistent with the results of the competing risk analysis of the whole samples, further supporting the robustness of our main conclusion. This may indicate that ELNs count is not a primary determinant in the mortality of cervical cancer patients. It means that the removal of a greater number of lymph nodes does not elevate the mortality risk for elderly patients with cervical cancer. Meanwhile, ELNs count exhibited a significant inverse association with non-cervical cancer mortality. Specifically, as the number of ELNs increased, the mortality rate from non-cervical cancers was observed to decrease. Non-cervical cancer deaths may encompass competing events such as cardiovascular diseases, infections, and other malignancies. ELN count can simultaneously reflect the thoroughness of surgery, the adequacy of pathological assessment, and the overall health status of the patient. Therefore, it still holds significant clinical value in elderly patients with cervical cancer. The results of the competing risk model suggest that ELN may more likely serve as a comprehensive prognostic marker rather than a single causal protective factor.

In addition to the ELNs, we also identified that FIGO stage, radiotherapy, and age were significantly associated with OS after multivariate regression analyses. These findings align with the conclusions of previous studies (12, 15).

To the best of our knowledge, nomograms have been utilized to forecast the survival outcomes for a variety of cancers (11, 18, 31). The nomogram, which evaluates risk by integrating and depicting the significance of various prognostic factors, has found application in clinical oncology assessment (32). To date, no studies have investigated whether ELNs are associated with the prognosis of elderly patients following cervical cancer surgery or can serve as a predictive factor. Therefore, prognostic factors derived from a multi-factor analysis of ELN count, FIGO staging, radiotherapy, and age were utilized as variables to develop a nomogram prediction model for elderly patients with cervical cancer. This model demonstrated high predictive accuracy and significant clinical utility in estimating survival rates at 1 year, 3 years, and 5 years post-surgery, as confirmed through rigorous validation. Therefore, it can predict the short-term risks while also provides reference value for long-term clinical decision-making.

Several limitations in this study should be acknowledged. Firstly, due to its retrospective design, certain biases were inevitable. Secondly, variability existed across different institutions within the SEER program regarding the number of lymph nodes removed and counted. An another limitation of the SEER database is its inability to differentiate between the scenarios leading to ELN = 0: a completed dissection with negative nodes versus no dissection performed. Additionally, specific details about LN stations and locations were unavailable for analysis. Thirdly, comprehensive data on surgical procedures, margin status, postoperative complications, recurrence status, and systemic therapy administration were not obtainable from the SEER database. Consequently, we could not investigate the correlation between progression free survival (PFS) or recurrence and ELN count. Furthermore, for certain patients, particularly those with FIGO stages III/IV, surgery might not have been the primary standard treatment as per current guidelines. These patients may have undergone neoadjuvant therapy or palliative surgery, potentially leading to inadequate or absent nodal sampling and positive surgical margins, which can affect the accuracy of ELN counts. Although residual confounding cannot be completely ruled out, we have adopted reasonable statistical controls and believe that the research conclusions within the framework of SEER data are of reference value and should be regarded as an important supplement to clinical practice. While we adjusted for all available clinicopathological factors, residual confounding remains a possibility. Therefore, our findings regarding ELN count require validation in cohorts with more comprehensive clinical data and more prospective studies.

## 5 Conclusion

In this study, we have identified ELN count as a potentially useful prognostic marker associated with the OS of patients with cervical carcinoma whose ages were equal to or over 65. We have developed a nomogram-based prediction model that demonstrates high predictive accuracy for 12-, 36-, and 60-month survival rates. This model can be a valuable tool for prognostic prediction in elderly patients with cervical cancer.

## Data Availability

The raw data supporting the conclusions of this article will be made available by the authors, without undue reservation.

## Footnotes

1
https://www.r-project.org/

## Supplementary material

SUPPLEMENTARY FIGURE S1 E-value analysis for the association of ELN count with overall survival.
