# Supplementary figures and images for: Investigating the prognostic significance of examined lymph node count in elderly women with cervical carcinoma: a SEER population-based study
Source: Front Med (Lausanne). 2025 Nov 25;12:1619214. doi: 10.3389/fmed.2025.1619214 (PMC12658318; doi:10.3389/fmed.2025.1619214)

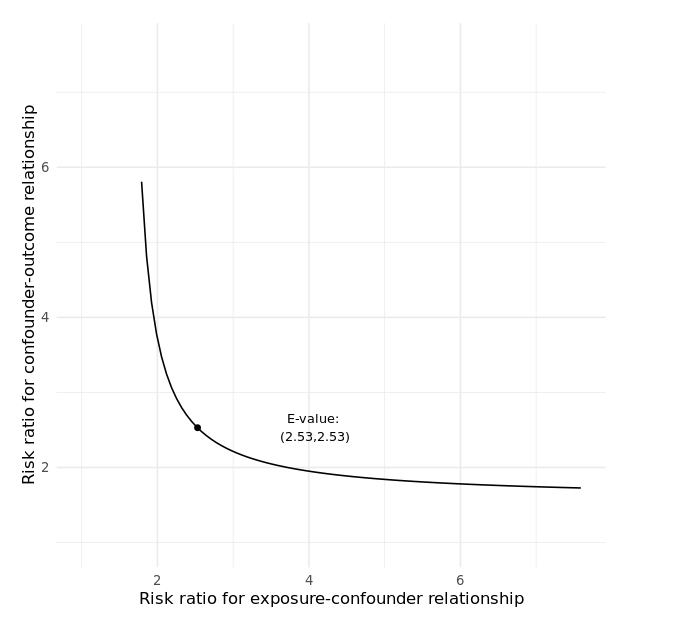

Supplement: SUPPLEMENTARY FIGURE S1 — E-value analysis for the association of ELN count with overall survival. [file Image_1.tif]
